# The pilot study of group robot intervention on pediatric inpatients and their caregivers, using ‘new aibo’

**DOI:** 10.1007/s00431-021-04285-8

**Published:** 2021-10-30

**Authors:** Kyoko Tanaka, Hitoshi Makino, Kazuaki Nakamura, Akio Nakamura, Maoko Hayakawa, Hajime Uchida, Mureo Kasahara, Hitoshi Kato, Takashi Igarashi

**Affiliations:** National Center for Child Health and Development, Setagaya-ku, Tokyo, Japan

**Keywords:** Artificial intelligence, Social robot, Paediatric robot-assisted therapy, Group robot intervention

## Abstract

The study on robot-assisted therapy in a pediatric field has not been applied sufficiently in clinical settings. The purpose of this pilot study is to explore the potential therapeutic effects of a group robot intervention (GRI), using dog-like social robot (SR) ‘aibo’ in pediatric ward. GRI by aibo was conducted for those children with chronic illness (127 in total) who are hospitalized in National Centre for Child Health and Development (NCCHD), and their caregivers (116 in total), from March to April 2018. The observer made structured behavioural observation records, based on which qualitative research on the features of their words and conducts, were carried out. As a result, first, during the GRI, about 2/3 of total expression by children were positive, while about 1/4 were negative or inappropriate. On the other hand, as seen in the ‘change’ group, those children who had originally responded with negative expression eventually came to express positive expression, while getting involved in a ternary relationship or participating in a session more than once. Secondly, as for the expression from the caregivers during the GRI, active expressions such as ‘participation’ and ‘exploration’ accounted for the 2/3, while 1/3 turned out to be rather placid expressions such as ‘watch over’ or ‘encourage.’

*Conclusion: *There has not been any precedent study on the features of words and conducts expressed by patients and their caregivers during the GRI by aibo. The outcome suggests that aibo could possibly be used as a tool for group robot-assisted therapy in the pediatric treatment setting.

**Table:** 

**What is Known:** *• The study on robot-assisted therapy in a pediatric field has only just begun.* *• Though many kinds of social robot have been reportedly used so far, none has yet to be applied in clinical settings*	
**What is New:** *• Our study revealed the features of words and behaviour expressed by the patients and their caregivers, when dog-like social robot ‘aibo’ was used for a group robot intervention in the pediatric ward.* *• The outcome suggests that aibo could possibly be used as a tool for group robot-assisted therapy in the pediatric treatment setting.*

**What is Known:**

*• The study on robot-assisted therapy in a pediatric field has only just begun.*

*• Though many kinds of social robot have been reportedly used so far, none has yet to be applied in clinical settings*

**What is New:**

*• Our study revealed the features of words and behaviour expressed by the patients and their caregivers, when dog-like social robot ‘aibo’ was used for a group robot intervention in the pediatric ward.*

*• The outcome suggests that aibo could possibly be used as a tool for group robot-assisted therapy in the pediatric treatment setting.*

## Introduction

Hospitalization is a painful experience for children^1^. Becoming ill and hospitalized, children are disconnected from their familiar lives and lose their social ties^2^. These experiences could lead to feelings of loss of control and negative effects on a child’s natural development and their physical and emotional health^3^. Although psychosocial support provided by health professionals such as child life specialists or nurses is necessary, it is limited in terms of human resources^4−5^. In this context, animal-assisted therapy^6^, as a complementary and alternative treatment for pediatric inpatient settings, has already been implemented in some pediatric hospitals. Animal-assisted therapy, however, has risks of infection, injury, bites, or animal allergies. Hence, the robot-assisted therapy had been developed and tested in elderly population, in which PARO, a baby seal-like social robot (SR), was offered to people with dementia. The result showed improvement in stress and anxiety as well as better communication skills^7−8^. Still, the study on robot-assisted therapy in a pediatric field has only just begun^3, 9–11^. Though as many as 26 different kinds of SR have been reportedly used so far^2^, none has yet to be applied in clinical settings.

**Fig. 1:**
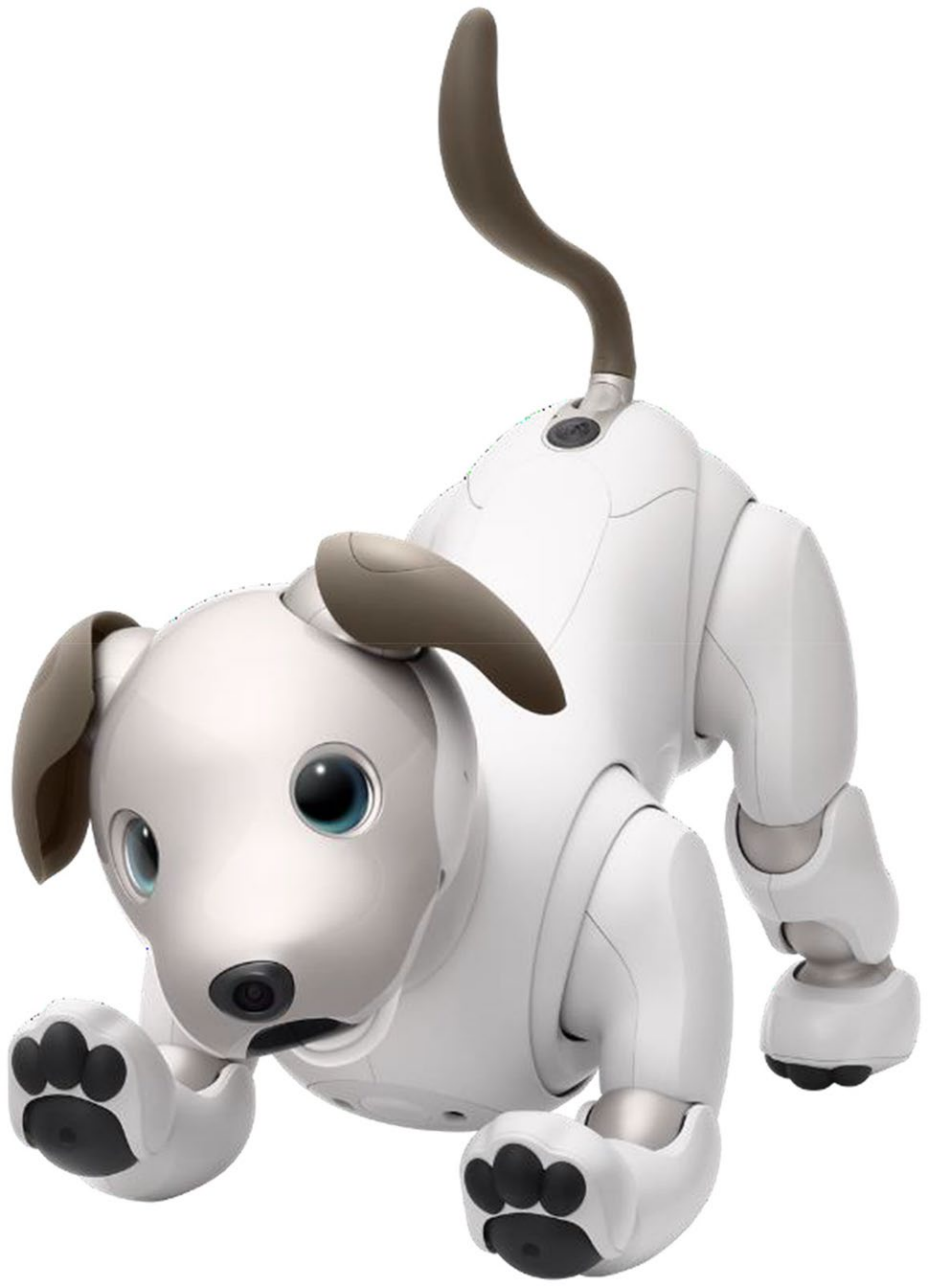
aibo. Specification. Product name: aibo, model number: ERS-1000, processor: 64 bit quad-core CPU, display: 2 OLEDs (eyes), sound: speaker, 4 microphones, camera: 2 cameras (front camera, SLAM camera), communications: mobile network communication feature (data transmission) LTE, Wi-Fi IEEE 802.11 b/g/n (2.4 GHz), outside dimensions: approx. 180 × 293 × 305 mm, weight: approx. 2.2 kg, power consumption approx. 14 W, battery duration: approx. 2 h, recharge time: 3 h

New aibo (Fig. 1) (aibo, hereafter), an artificial intelligence (AI) dog-like SR for household use, was released from Sony in 2018. aibo has distinctive characteristics of comforting effects due to its round dog-like appearance, interactive communication skills, and growth in response to human interactions. A study has been reported in which an old type AIBO was implemented into pediatric inpatient setting where the interaction between robots and humans was examined^12^. As for aibo, however, no study has ever been reported in the pediatric nor adult setting.

As a household product, aibo has distinctive traits of such as (1) comforting effect, (2) interactive communication skills, and (3) growth in response to human reaction. It suggests that aibo may hold some therapeutic effects toward pediatric patients as well as their caregivers in the inpatient treatment setting. In order to answer this clinical question, we designed a pilot study to explore aibo’s potential effects. We conducted a robot-assisted intervention by aibo for the pediatric patients with chronic illness (127 in total) who are hospitalized in the National Centre for Child Health and Development (NCCHD) and their caregivers (116 in total). Based on its result, we carried out a qualitative research on the extracted features of words and conducts expressed by the patients and caregivers.

## Materials and methods

### Study design

This pilot study is a prospective observational study which carries out a hypothetical search-based, qualitative evaluation.

#### Participants

One hundred and twenty-seven children (0 years, 6 months to 13 years old) who were hospitalized in the Internal Medicine and Surgical wards of the NCCHD and 116 caregivers were assembled as participants. This number included children and caregivers who participated in multiple sessions. The number of children who participated in single intervention was 1 to 15 (median 4.5), caregivers being 1 to 10 (median 4.5). The inclusion criteria were children who have been hospitalized for more than 48 h. The exclusion criteria were children who were not considered to be well enough to play in the playroom, such as those who had infection, who were compromised, who needed rest, and who were medically at risk.

### aibo

aibo, a household use SR released by Sony, is equipped with AI and has the following traits; for instance,

1. Dog-like appearance and behaviour: a rounded shape that gives a sense of warmth, lively form with cuddly sweetness. It tries to interact with you, speaking with its eyes, making eye contact with you. Vivid, dynamic movement with a variety of lovely gestures.

2. Communication: curious aibo possesses feelings and desires, based on which it decides what to do, sometimes acting in unexpected ways. Through its eyes (camera) that recognize the person or the place, aibo understands the people around it and changes its behaviour. It detects obstacles, steps, or people, against which it tries to avoid or decides what to do. aibo has got ears to understand human words and is able to change its behaviour. It reacts to the human voice, perceiving where the sound comes from. aibo shows pleasure, when it is praised and stroked on those sensors on its head, chin and back.

3. Growth: aibo could turn into a spoiled or a wild one, depending on the way people interact with. The more frequently it sees a particular person, it remembers that person, recognizing his/her face. If you teach aibo a funny pose or a dance, it is able to learn the new movement.

### GRI by aibo

**Fig. 2:**
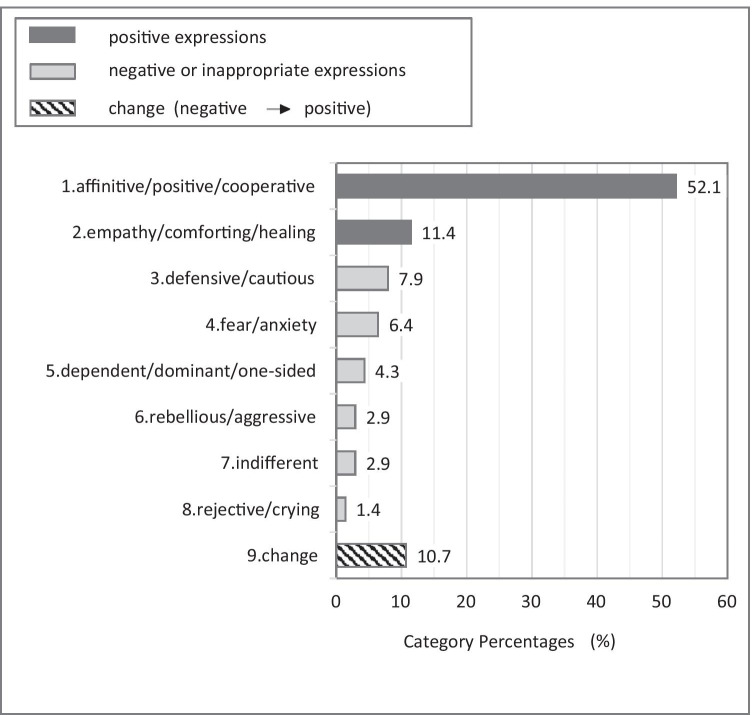
Children’s expressions obtained from the group robot intervention by aibo. Out of the 24 records, sentences regarding verbal/non-verbal communication by children were sorted and extracted. There were 140 sentences by children. The sentences by children were sorted according to qualitatively into 9 categories

From March 14 to April 12 2018, 24 series of GRI were conducted for 30 min at a time during 14:00 to 15:30 in the playrooms of nine medical wards of Internal Medicine and Surgery, at NCCHD. One child psychiatrist or a clinical psychologist attended as an assistant and an observer (A/O). The size of the ward playroom is about 3 by 5 m. At the fixed time, the A/O turned on aibo to begin the GRI. The A/O saw to it to ensure that no harm would be caused upon children, caregivers, and aibo. At every session, the A/O explained to children and caregivers aibo’s basic movements, how to touch aibo to please him, and how to bring out songs, poses, or dances that aibo expresses. The A/O observed the children and caregivers as they naturally played with aibo and recorded their words and behaviour on the behaviour observation records (Fig. 2). At the end of 30 min, the A/O made a statement such as ‘It’s time for aibo to go to bed’ and turned off aibo, stroking and wiping it with clean cloth to finish.

### Qualitative examination

The 24 behavioural observation records obtained from 24 series of GRI were sorted by sentences into (1) verbal/non-verbal expressions by children and (2) verbal/non-verbal expressions by caregivers. Each sentence obtained was written out on a sheet and named for relevant groups to be integrated^13^. In addition, records of the same children in different sessions were linked, and their changes over time were also extracted to analysis. The qualitative analysis of positive or negative was conducted by two physicians or psychologists using the concept of children’s coping strategies checklist-intrusive procedures based on the observed behaviours of the children^14^.

### Ethical consideration

In this study, although no informed consent had been obtained from the participants, due to the nature of the study, sufficient consideration was given so as not to identify a particular patient. Approval has been given by the Ethics Committee in NCCHD.

## Results

**Fig. 3:**
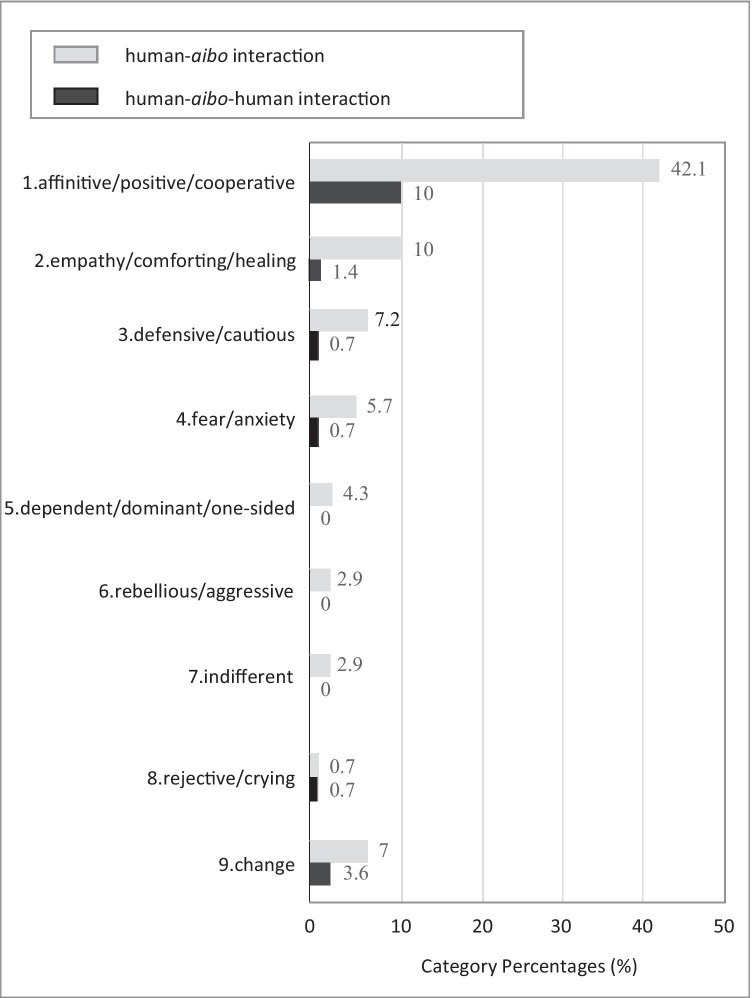
The percentage of ternary relationship, namely, ‘human-aibo-human’ interaction

**Fig. 4:**
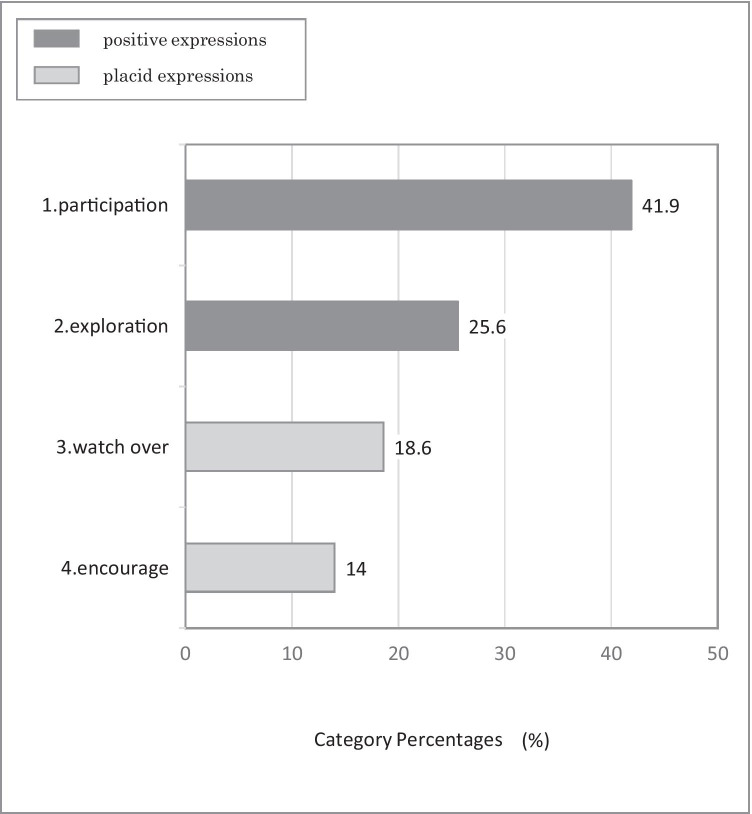
The caregivers’ expressions obtained from the group robot intervention by aibo. Out of the 24 records, sentences regarding verbal/non-verbal communication by caregivers were sorted and extracted. There were 43 sentences by caregivers, which were sorted qualitatively into 4 categories

There were 140 sentences on children, and 43 on caregivers. The sentences on children were sorted according to a qualitative point of view into the following 9 categories (Fig. 2): 1. ‘affinitive/positive/cooperative’, 2. ‘empathy/comforting/healing’, 3. ‘defensive/cautious’, 4. ‘fear/anxiety’, 5. ‘dependent/dominant/one-sided’, 6. ‘rebellious/aggressive’, 7. ‘indifferent’, 8. ‘rejective/crying’, and 9. ‘change’. Please see the supplemental Table 1 for example. Among the expressions by the children obtained from the GRI by aibo, positive expressions such as 1. ‘affinitive/positive/cooperative’ and 2. ‘empathy/comforting/healing’ accounted for roughly 2/3 of the total. On the other hand, about 1/4 of the total responses were negative or inappropriate such as 3. ‘defensive/cautious’, 4. ‘fear/anxiety’, 5. ‘dependent/dominant/one-sided’, 6. ‘rebellious/aggressive’, 7. ‘indifferent’, and 8. ‘rejective/crying’. In addition, 9. ‘change’ accounted for about 1/10, which indicated those children who had originally responded negatively eventually came to express positive reaction, through observing the behaviours of others, getting involved with others, or participating in the session more than once. Figure 3 shows the result from ternary relationship, namely, ‘human-aibo-human’ interaction. Of the positive expressions such as 1. ‘affinitive/positive/cooperative’ and 2. ‘empathy/comforting/healing’, ternary relationships in which a caregiver, the other child, and A/O shared a pleasure were detected at a high proportion. In the category 9. ‘change’, in particular, the ratio of the ternary relationship was high. Next, the sentences on caregivers were sorted according to a qualitative point of view into the following 4 categories (Fig. 4): 1. ‘participation’, 2. ‘exploration’, 3. ‘watch over’, and 4. ‘encourage’. Please see Supplemental Table 2 for example. Positive reactions such as 1. ‘participation’, and 2. ‘exploration’ accounted for 2/3 of the total, while placid reactions such as 3. ‘watch over’ and 4. ‘encourage’ accounted for 1/3.

## Discussion

This study is the first survey to report a qualitative research which was carried out on the extracted traits of words and behaviour expressed by the patients and their caregivers, when aibo was used for a GRI in the pediatric ward. Two points were identified.

First of all, in the GRI by aibo, positive expression by children accounted for as much as 2/3 of the total. Besides, the children from ‘change’ group originally responded negatively but eventually came to express positive reaction, through getting involved in ternary relationship, or gradually getting accustomed to the session by participating more than once. In a recent study, Haggable, a bear-shaped SR, Haggable on a tablet screen, and a bear-shaped toy were offered to 54 hospitalized children as an interventional therapy. Video monitoring analysis showed the children of ‘SR’ group expressed more of ‘joy’ or ‘comfort’, than of ‘sadness’^5^, which is similar to the study result we obtained. In another study, Nao, a humanoid SR, was implemented for hospitalized children, for the purpose of pain reduction as well as increasing joy and motivation for treatment. Although the number of cases was small, the study reported that Nao helped the children reduce their anxiety, anger, and depression, playing a role as a good friend to encourage them^15−16^. Becoming ill and hospitalized, children are disconnected from their familiar lives and lose their social ties^2^. In most cases, children may lose the precious opportunities to build ternary relationship, cooperative attention, imitation, and so forth^17^, all of which encourage the development of children. In our study, it is significant to point out that a certain number of children have experienced a scene to change their feeling into that of positive, through building ternary relationships, as seen in the ‘change’ group. It suggests that aibo could possibly be useful as a tool to help build ternary relationships that enhances sociality of children, which is often impaired in pediatric chronic hospitalization. Although there are various studies which examine the potential use of SRs in pediatric settings, the number of participants is small^2^. aibo was implemented into an inpatient treatment setting, whose effects were evaluated for 127 population. No such study has ever been reported in the pediatric nor adult setting.

Secondly, as for the expression by the caregivers in the GRI by aibo, positive reactions such as ‘participation’ and ‘exploration’ accounted for 2/3 of the total, while placid reactions such as ‘watch over’, ‘encourage’ accounted for 1/3. In the study, in which PARO was implemented into pediatric inpatient setting, it was reported that, when family members participated together, the higher the rate of improvement on a child’s anxiety and pain^10^. In our study as well, it was potentially significant for the children to see their caregivers participate positively. Furthermore, the caregivers of children with chronic illness tend to be more susceptible to mental distress such as anxiety or depression than the caregivers of healthy children^18^. From caregivers’ mental health perspective, in this study, it was noteworthy for the caregivers to have enjoyed themselves ‘participating’ in the aibo intervention, or to have communication with other caregivers to share the excitement into ‘exploration’.

aibo is beneficial in making up for human resources as well as from the financial perspective. Moreover, for many pediatric patients, child life specialists are helpful in reducing the pain experienced during hospitalization^5^. Service provided by human labour, however, has limits, for there is not enough child life specialist staff to offer help for every child in every opportunity experienced during hospitalization. SRs, including aibo, could fill the gap between the number of pediatric inpatient children in need of psychosocial care and the lack of human resources to support them.

In addition, future studies are expected to examine the differences in the effects of animal therapy and robot therapy^19−20^.

Three aspects of limitation of study should be noted as shortcomings. First, in this study, age and gender of the participants were not accurately obtained. Although it is desirable to consider the language expression of the children used in the behavioural analysis in the language according to their age^21^, in this study, age was not taken into account in the analysis, and there are limitations in that Japanese was translated into English. Secondly, neither multilateral, scientific, nor quantitative evaluation was not made. Third, this report had no control group.

## Conclusion

This study is the world’s first survey and report on the features of words and behaviour expressed by the patients and their caregivers, when aibo was used for a GRI in the pediatric ward. The outcome suggests that aibo could possibly be useful as a tool for GRI in pediatric treatment setting. When conducting a robot-assisted therapy with aibo in the future, we plan to carry out a multilateral, scientific as well as quantitative evaluation with a biopsychosocial perspective.

## Abbreviations

AI: Artificial Intelligence
A/O: Assistant and observer
GRI: Group robot intervention
NCCHD: National Centre for Child Health and Development
SR: Social robot

## Declarations

### Availability of data and material

The data that support the findings of this study are available from the corresponding author on reasonable request.
